# The neuroprotective effects of icariin on ageing, various neurological, neuropsychiatric disorders, and brain injury induced by radiation exposure

**DOI:** 10.18632/aging.203893

**Published:** 2022-02-14

**Authors:** Ling Rui Li, Gautam Sethi, Xing Zhang, Cui Liu Liu, Yan Huang, Qun Liu, Bo Xu Ren, Feng Ru Tang

**Affiliations:** 1 The School of Basic Medicine, Health Science Center, Yangtze University, Jingzhou 434023, Hubei, China; 2 Department of Pharmacology, Yong Loo Lin School of Medicine, National University of Singapore, Singapore 117600, Singapore; 3 Radiation Physiology Lab, Singapore Nuclear Research and Safety Initiative, National University of Singapore, Singapore 138602, Singapore

**Keywords:** icariin, oxidative stress, neuroinflammation, ageing, radio-neuro-protective effect

## Abstract

Epimedium brevicornum Maxim, a Traditional Chinese Medicine, has been used for the treatment of impotence, sinew and bone disorders, “painful impediment caused by wind-dampness,” numbness, spasms, hypertension, coronary heart disease, menopausal syndrome, bronchitis, and neurasthenia for many years in China. Recent animal experimental studies indicate that icariin, a major bioactive component of epimedium may effectively treat Alzheimer’s disease, cerebral ischemia, depression, Parkinson’s disease, multiple sclerosis, as well as delay ageing. Our recent study also suggested that epimedium extract could exhibit radio-neuro-protective effects and prevent ionizing radiation-induced impairment of neurogenesis. This paper reviewed the pharmacodynamics of icariin in treating different neurodegenerative and neuropsychiatric diseases, ageing, and radiation-induced brain damage. The relevant molecular mechanisms and its anti-neuroinflammatory, anti-apoptotic, anti-oxidant, as well as pro-neurogenesis roles were also discussed.

## INTRODUCTION

**Figure 1:**
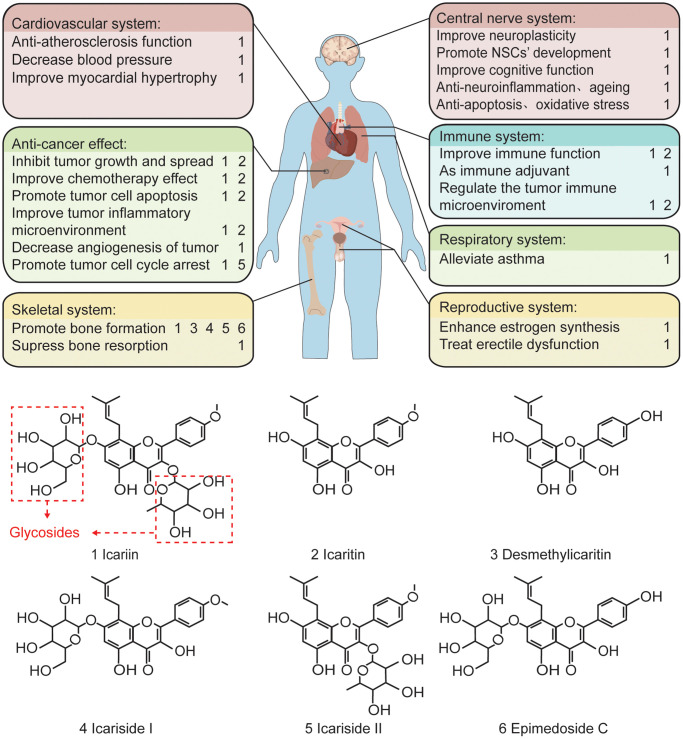
**The pharmacological effects of the main components of epimedium and the related chemical formula.** In terms of function, icariin plays a protective and anti-tumor role in various cardiovascular, skeletal, central nervous, immune, respiratory, and reproductive systems. Structurally, icariin has a chemical structure of glycosides, which is one of the reasons for its low oral bioavailability.

Icariin (molecular formula: C33H40O15, molecular weight: 676.67 g/mol) [1], a prenylated flavonoid glycoside, is derived from the Chinese herb Epimedium sagittatum [2] or yin yang huo [3]. Epimedium is a Traditional Chinese Medicine (TCM) used for thousands of years [1]. In Asian countries, it is used as a traditional tonic agent for ageing, male sexual dysfunction, and major human body systems [4]. The essential components of epimedium include icariin, icaritin, desmethylicaritin, icariside I, and icariside II. As a primary component of epimedium, icariin has many pharmacological effects, including treatment of impotence [5], ameliorating sexual dysfunction, promoting estrogen synthesis [6], and osteoporosis. Moreover, icariin can exhibit both immunomodulatory and anti-inflammatory effects [7], antioxidant activity [4], anti-ageing [8], improvement of cardiovascular function, in addition to be anti-bacterial [9] and anti-tumor [10–13]. It has been used in the management of hypertension, coronary heart disease, osteoporosis, menopausal syndrome, rheumatism, neurasthenia, bronchitis, and hypogonadism (Figure 1) [14]. Moreover, recent studies have also suggested that icariin produces neuroprotective effects and can significantly increase the viability of hippocampal neurons treated with corticotropin releasing hormone (CRH) for 24 h [15]. It alleviates the cognitive deficits in the senescence accelerated mouse prone 8 (SAMP8) Alzheimer’s disease (AD) animal model by inhibiting the formation of amyloid plaques through the downregulation of amyloid-beta 1-42 (Aβ1-42), suppression of neuronal death as well as promoting apoptosis by increasing the B-cell lymphoma 2 (Bcl-2)/Bax ratio [16]. It also protects sodium azide (NaN_3_)-induced neurotoxicity in PC12 cells by activating the phosphoinositide 3-kinase/protein kinase B/glycogen synthase kinase-3β (PI3K/Akt/GSK-3β) signaling pathway [17]. In this paper, we reviewed the pharmacological effects of icariin on different neurological and neuropsychiatric disorders, ionizing radiation-induced brain damage and their relevant molecular mechanisms.

## Pharmacodynamics of icariin in neurodegenerative diseases

Neurodegenerative diseases are incurable and patients are in debilitating conditions with progressive degeneration or death of nerve cells, which may cause problems with movement or mental functioning. Neurodegenerative diseases, including brain trauma, brain ischemia, AD, Parkinson’s disease (PD), multiple sclerosis (MS) and Prion disease, Motor neuron diseases (MND), Huntington’s disease (HD), Spinocerebellar ataxia (SCA), are caused by the loss of neurons or their myelin sheath [18]. The leading causes of neurodegeneration include oxidative stress, mitochondrial dysfunction, excitatory neurotoxicity, immune inflammation, and apoptosis [19]. Extensive studies have shown that icariin has anti-angiogenesis, anti-autophagy, anti-apoptosis, anti-inflammatory as well as anti-oxidative effects and promotes neurogenesis [1].

### AD

AD has an increasing prevalence globally because of the ageing of the global population [20–22]. The characteristic of AD includes memory deficits and cognitive decline which has brought serious economic and psychological burden to the patient family and society [23]. The neuropathological changes of AD include abnormal deposition of Aβ, neurofibrillary tangles, hypofunction of cholinergic neurons, loss of synapses, dendritic spines, and regionally specific neuronal apoptosis in the brain [24, 25]. So far, the pathogenesis of AD has not been accurately elucidated and there is no suitable drug to effectively prevent or reverse AD’s pathological process [26, 27]. Icariin has been found to cross the blood-brain barrier, exert a neuroprotective effect [28], and improve the spatial learning and memory ability in different animal models [25, 29]. It can effectively improve memory dysfunction by restoring atrophies of axons and dendrites even when amyloid β-induced neurite atrophy has already occurred in 5×FAD mouse model [30]. Aβ plaque is the most significant pathological marker of AD. Preventing or slowing down the formation of Aβ is an efficient therapy to AD development [31]. Aβ is produced in the brain by transmembrane amyloid precursor protein (APP) cleaved sequentially by β-site amyloid precursor protein cleaving enzyme 1 (BACE1) and γ-secretase complex [32]. Thus, inhibition of APP, BACE1 and γ-secretase expression may be closely related to the decrease of Aβ production. A number of previous studies has shown that icariin enhances the ability of spatial learning and memory by reducing the Aβ1-40 and beta-secretase and increasing superoxide dismutase-2 in Aβ25-35-induced rat model [33]. Icariin decreased the expression of APP, BACE1 and reduced Aβ deposition in APP/PS1 Tg mouse model [34]. Moreover, icariin could significantly decrease the expression of APP and Aβ and increase neurogenesis in Tg2576 mouse model [26]. These findings further support the important role of icariin in the prevention and treatment of AD.

Accumulating evidences suggest that hyperphosphorylation of Tau protein is an early event in the development of AD, which leads to neurotoxic effects and ultimately neurodegeneration [35]. Therapy to inhibit tau hyperphosphorylation has gradually become a treatment to prevent the development of AD [36]. GSK-3β is one of the significant tau kinases in tau protein hyperphosphorylation [37]. The GSK-3β expression level is higher in APP transgenic cultures, and thereby suppressing GSK-3β expression may reduce Aβ-induced toxicity of hyperphosphorylated tau. Icariin exerts neuroprotective effects on PC12 cells treated with Aβ25-35 by inhibiting hyperphosphorylation of tau through suppressing the PI3K/Akt-dependent GSK-3β signaling pathway [35]. Therefore, icariin may be a candidate drug for the treatment of AD and other tau protein abnormalities.

Vascular risk factors and cerebrovascular injury may increase the risk of AD genesis [31]. Long-term chronic cerebral hyperperfusion is a key factor in the development of degenerative brain lesions and AD-related pathology [38]. Icariin can alleviate cognitive impairment in permanent bilateral ligation of the common carotid arteries (2-VO) rat model [39]. Aluminum reduces the learning and memory ability by inducing brain damage in the rat model leading to the pathogenesis of AD [40]. Icariin improves the learning and memory impairment of rats induced by aluminum and reverses the decrease of superoxide dismutase (SOD) activity and the increase of malondialdehyde (MDA) level in the hippocampus of rats under aluminum exposure [41]. In animal models of AD, abnormal brain nitric oxide/soluble guanylate cyclase/ cyclic guanosine monophosphate/protein kinase G/cyclic adenine monophosphate responsive element binding protein (NO/sGC/cGMP/PKG/CREB) signaling pathway is observed [42]. Phosphodiesterase 5 (PDE5) is a cGMP degrading enzyme, and PDE5 inhibitors can improve synaptic plasticity and memory function by increasing cGMP and thereby activating NO/sGC/cGMP/PKG/CREB signaling pathway [42]. In APP/PS1 Tg mouse model, icariin improved the learning and memory functions by inhibiting PDE5 activity and activating NO/cGMP signaling [43]. These results indicate that icariin may act as a potent therapeutic agent against AD. Further studies are still needed to analyze the therapeutic potential of icariin in large animal model and patients with AD.

### PD

PD, a progressive neurodegenerative disorder with the loss of dopaminergic neurons in the midbrain substantia nigra [44] is characterized by slow movement, static tremor, rigidity, and posture instability [45, 46]. PD patients also show autonomic, cognitive, psychiatric and sleep dysfunctions [47, 48]. The main etiology of PD includes gene mutations and 1-methyl-4-phenyl-1,2,3,6- tetrahydropyridine (MPTP) exposure. PD may also be related to heredity, environment and ageing [48]. Due to the complex pathogenesis of PD, no ideal therapeutic drug with few side effects has been developed so far. Some traditional herbs show beneficial effects on PD [49], which have led to active research in this field. For instance, icariin was found to reduce the loss of dopaminergic (DA) neurons in the substantia nigra pars compacta (SNpc), suppress the expression of Bax and Caspase-3, increase the levels of Bcl-2 in the striatum in MPTP-induced PD mouse model [50]. Chen et al. (2016) confirmed that PI3K/Akt and MEK/extracellular signal-regulated kinase (ERK) signaling pathways were involved in the neuroprotective effect of icariin in both *in vivo* and *in vitro* models [50]. These results suggest that icariin may be one of the candidate herbs for PD treatment.

It has been established that neuroinflammation caused by glial activation is related to the pathogenesis of PD [51]. Icariin has been shown to protect DA neurons from lipopolysaccharide/6-hydroxydopamine (LPS/6-OHDA) damage, improve motor performance, reduce microglial activation, decrease tumor necrosis factor-α (TNF-α), interleukin-1β (IL-1β), and NO production by inhibiting nuclear factor-ĸB (NF-ĸB) signaling pathway under both *in vitro* and *in vivo* settings [52]. Although NF-ĸB is a key factor involved in neuroinflammation, other factors related to neuroinflammation cannot be ignored. Nuclear factor erythroid 2 related factor 2 (Nrf2) has been proved to have anti-inflammatory properties [53]. A number of recent studies has demonstrated that icariin protects DA neurons against 6-OHDA and suppresses the glia cells-elicited neuroinflammation through activating Nrf2 signaling [54]. The above studies show that icariin may reduce neuroinflammation by inhibiting NF-ĸB pathway or activating Nrf2 signaling pathway. Further study in different animal models of PD may be needed in order to imitate different features of PD patients, so that more effective drugs could be developed to control patient’s symptoms.

### Ischemia and Stroke

Cerebral ischemia may be caused by stroke, anemia, and cardiovascular diseases leading to neuronal damage as well as impairment of brain functions [55]. The molecular mechanisms involved may include glutamate-mediated/induced excitotoxicity, reactive oxygen species production, DNA damage, regulation of pro-apoptotic factors [56]. So far, there is still no drug to control ischemia-induced brain damage effectively. Some bioactive components from herbs, such as icariin, appear to have neuroprotective roles after ischemia [55]. In the rat brain ischemia-reperfusion model, icariin effectively prevents neuronal damage and improves animal cognition [57, 58].

Cerebral ischemia activates microglia and induces microglia to produce inflammatory mediators to aggravate brain tissue damage [59]. In a cerebral ischemia-reperfusion (I/R) injury model induced by middle cerebral artery occlusion (MACO), Xiong et al. (2016) reported that icariin significantly improved I/R-inducibility and reduced the infarct volume by its anti-inflammation effect through markedly down-regulating the levels of IL-1β and TGF-β1 proteins by inhibition of NF-ĸB activation. Furthermore, icariin was found to increase both peroxisome proliferator-activated receptors α (PPARα) and PPARγ protein expression in the brain tissue. These results indicated that icariin may prevent brain ischemic injury by targeting on both PPARα and PPARγ [60]. Icariin may also significantly improve the learning and memory by activation of cholinergic system and the scavenging of oxygen free radicals [61]. It reduces 6-OHDA-induced neurotoxicity by stimulating Nrf2 antioxidative signaling pathways in PC12 cells [19], and protects mouse primary cortical neurons after oxygen and glucose deprivation (OGD), which may be partially associated with the increase of sirtuin 1 (SIRT1) via the activation of mitogen-activated protein kinase (MAPK)/p38 signaling pathway [62]. Moreover, icariin also reduces brain ischemic injury by upregulating SIRT1-dependent peroxisome proliferator-activated receptor γ coactivator-1α (PGC-1α) expression [63]. These studies strongly suggest that icariin is a promising neuroprotectant to treat ischemic- or stroke-induced brain injury and subsequent neurocognitive impairment.

### Depression

**Figure 2:**
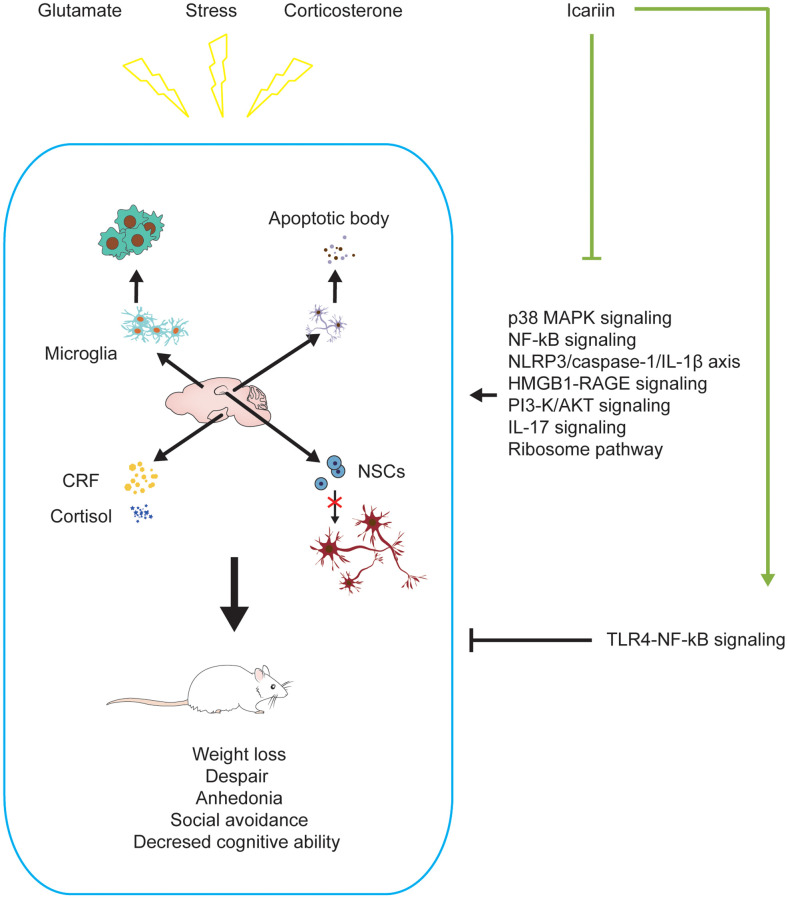
**Icariin alleviates depression-like changes induced by stress, corticosterone, and glutamate in depressive models.** Icariin can downregulate the levels of apoptosis, neuroinflammation, CRF, cortisol and promote neurogenesis in the brain to improve the depression-like behaviors. The mechanisms may be inhibiting P38 MAPK, NF-ĸB, HMGB1-RAGE, PI3-K/AKT, IL-17, ribosome signaling pathways, NLRP3/Caspase-1/IL-1β axis, and activating TLR4-NF-ĸB signaling pathway.

Depression has once been considered a disease often suffered by middle-aged women. It now becomes a public health problem that affects people of different ages and economic backgrounds [64]. Patients with depression have severe mood disorders; such as persistent feeling of sadness, loss of interest and concentration [65]. In some cases, patients may hurt themselves, causing an increased rate of disability and suicide [66, 67]. Depression limits the victim’s psychosocial functions and reduces the quality of life [68]. Psychological and drug treatments have been used to control syndromes. For mild-to-moderate depression, psychotherapeutic intervention is a preferred method [69]. Antidepressants have been the primary treatment for depression. So far, the pathophysiology of depression is still unclear. It may be linked to a decline in the function of the monoaminergic neurotransmitters (serotonin, norepinephrine, dopamine, or combined) in the brain, as antidepressants targeted on these neurotransmitters can effectively mitigate the functional impairments [70]. However, the clinical efficacy of these drugs is rather limited with some side effects [55, 67, 68]. Icariin has been considered a promising antidepressant with minimum adverse effects [55]. In the rodent models of depression, icariin decreased immobility time in the forced swim test and tail suspension test [28, 71, 72], indicating its potential anti-depressive effects. Notably, icariin reversed the elevated oxidase monoamine oxidase A and B levels and the decreased monoamine neurotransmitter levels in the brain caused by the forced swim test [72]. In addition, icariin was recently reported to improve hippocampal neurogenesis in a rat model of depression [73] (Figure 2).

The association between the abnormalities of the hypothalamic-pituitary-adrenal (HPA) axis and depression has been established in the previous study [74]. The anti- depressant-like activity of icariin may be linked to its regulation on the central neuroendocrine system or the abnormal HPA axis [55]. In rats exposed to chronic mild stress (CMS), Pan et al. (2010) found that icariin could down-regulate the levels of serum corticotropin-releasing factor (CRF) and corticosterone (CORT) as well as decrease the levels of CRF mRNA and protein in the hypothalamus [75]. These results suggested that icariin could reverse CRF system hyperactivity, which was further proved in the subsequent studies on rats exposed to chronic unpredictable mild stress (CUMS) [76]. Moreover, icariin has been shown to decrease FK506 binding protein 5 (FKBP5) mRNA levels in serum and glucocorticoid-inducible kinase 1 (SGK-1) in the hippocampus and prefrontal cortex. Icariin partially reversed the upregulated expression of nuclear GR in the prefrontal cortex and that of FKBP5 in the hippocampus. It may therefore restore the negative feedback function of the HPA axis and produce antidepressant-like effects [77].

High concentrations of CORT induce cell apoptosis, leading to the neuronal damage and subsequent depression-like behavior. Accumulated evidence indicates that the antidepressant-like activity of icariin may be related to its anti-CORT-induced apoptosis. In primary cultured rat hippocampal neurons, icariin produces a neuroprotective effect against CORT-induced apoptosis and mitochondrial dysfunction through blockade of p38 MAPK phosphorylation [78]. In addition, icariin pretreatment restores CORT-induced abnormity in caspase-3 activity, intracellular reactive oxygen species and superoxide dismutase activity, mitochondrial membrane potential, and the loss of neurons by activating the PI3K/Akt pathway in primary cultured hypothalamic neurons [79].

It is important to highlight that neuroinflammation may play an important role in the development of depression [80, 81]. LPS induces pro-inflammatory factors in the immune cells of animals, such as TNF-α, IL-1β, and interleukin-6 (IL-6), which in turn cause depression-like behaviors [81, 82]. The possible mechanisms involved may include the increase of oxidative stress [82, 83] and the activation of the brain-derived neurotrophic factor (BDNF) signaling pathway [84]. Recent studies have suggested the importance of icariin as the anti-neuroinflammatory agent that produces significant antidepressant effects. In neuron-microglia co-culture system, icariin decreased LPS-induced pro-inflammatory mediators such as prostaglandin E (PGE)-2, NO, TNF-α, IL-1β, IL-6, and cyclooxygenase-2 (COX-2), in activated microglia by the inhibition of JNK/p38 MAPK and TGF-β activated kinase-1 (TAK1)/IκB kinase (IKK)/NF-κB signaling pathways [85]. In rats with LPS-induced brain dysfunction, icariin reduces escape latency and the searching distance in the Morris water maze (MWM) test, suggesting improved spatial learning and memory. Meanwhile, it decreases brain COX-2, IL-1β, and TNF-α levels [86]. In an unpredictable chronic mild stress model of depression, icariin restrains activation of the nod-like receptor protein 3 (NLRP3) inflammasome and elevation of IL-1β and caspase-1 protein levels in the rats’ hippocampus, as well as activation of NF-ĸB signaling and increase of oxidative-nitrosative stress markers [28]. Icariin has also been shown to attenuate immobility in the forced swim test and normalize hippocampal BDNF levels in a rat model of CORT-induced depression [87]. In a social defeat mouse model, icariin reverses the translocation of High mobility group protein box 1 (HMGB1) from the nucleus to the cytoplasm, which has been found to be involved in stress-induced inflammation [88]. These studies strongly suggest that the neuroprotective effects of icariin may be partially related to its anti-neuroinflammatory effect.

Icariin may also produce an antidepressant-like effect by affecting neurotransmission system. In the prenatal restraint stress (PRS)-induced depression rat model, icariin relieves PRS-induced depressive-like behavior accompanied by a decrease in excitatory amino acid transporter 2 (EAAT2), metabotropic glutamate receptor 1 and 5 (mGluR1 and mGluR5) expressions in the hippocampus [14]. In rats with depression induced by CORT, glucose metabolism decreases in the brain, and the depression-like behaviors are improved when the glucose metabolism level is increased after treatment with icariin [89, 90]. In other models of depression, abnormal amino acid and lipid metabolism were observed in the hippocampus, cortex, and thalamus [91, 92]. Icariin increased amino acid, glucose, and lipid metabolism in the rat model of CORT-induced depression [87]. These findings suggest that icariin may be used as a candidate drug to treat depression-like behavior by regulating abnormal metabolic pathways.

### MS

MS is an autoimmune disease with inflammation, demyelination, and axon damage as the main neuropathological changes in the CNS [93]. It is common in young people and has many main clinical manifestations that appear singly or in combination, such as vision loss or diplopia, limb weakness or sensory loss, or ataxia [94]. At present, the specific pathogenesis of MS is unknown. Several important factors such as the Epstein-Barr virus, smoking, ultraviolet radiation B (UVB), vitamin D, and genetic background have been reported to be involved in pathogenesis of the disease [93]. Clinical treatment of MS is limited to symptomatic relief, hormone therapy, and immunosuppressant medicine may cause severe complications [95, 96]. In a commonly used experimental autoimmune encephalomyelitis (EAE) MS model, icariin and methylprednisolone (a CORT) produces a synergistic effect to enhance anti-inflammatory functions [96]. In addition, icariin promotes myelin regeneration and axonal repair during the remission period by increasing oligodendrocyte numbers and nerve growth factor levels in the cuprizone-induced acute demyelination model [97].

The central nervous system inflammatory infiltration by T cells is accompanied by microglial activation in multiple sclerosis, leading to oligodendrocyte reduction and demyelination [93]. Icariin reduces the number of Th1 and Th17 cells in spleen and lymph nodes, Th17 cells in CNS in EAE mouse model, and inhibits T cell proliferation and Th1 and Th17 cell differentiation *in vitro* [98]. Icariin attenuates clinical scores, improves demyelination, and reduces inflammation by inhibiting the activation of CNS inflammatory pathways such as the NF-ĸB pathway in another EAE mouse model of MS [99]. However, Wei et al. (2016) reported that icariin only decreased CORT but not IL-17 level in the EAE mouse model [95]. The difference may be due to the MS model used by different laboratories and further studies are still needed to clarify the inconsistency of results among different research groups.

There are evidences to suggest that the regulation of the HPA axis is related to the progression, severity, and prognosis of MS [100]. Glucocorticoid is the end product of the HPA axis, CORT is the main glucocorticoid in rodents, and its level partly reflects the activity of the HPA axis [101]. Because of the anti-inflammatory effects of glucocorticoids, activation of the HPA axis in MS plays a neuroprotective role. However, hyperactivity of the HPA axis is also associated with severe neurodegeneration, and low activity of the HPA axis seems to be consistent with a more active MS lesion [100, 101]. In EAE mouse models, icariin produces synergistic effects with MP to reduce IL-17 and CORT levels in serum [96]. In addition, icariin alone reduces the mean clinical score of EAE mice with significantly reduced serum CORT levels [95]. These experimental data indicate that icariin may serve as a novel drug to treat MS.

## Pharmacodynamics of icariin on ageing

**Figure 3:**
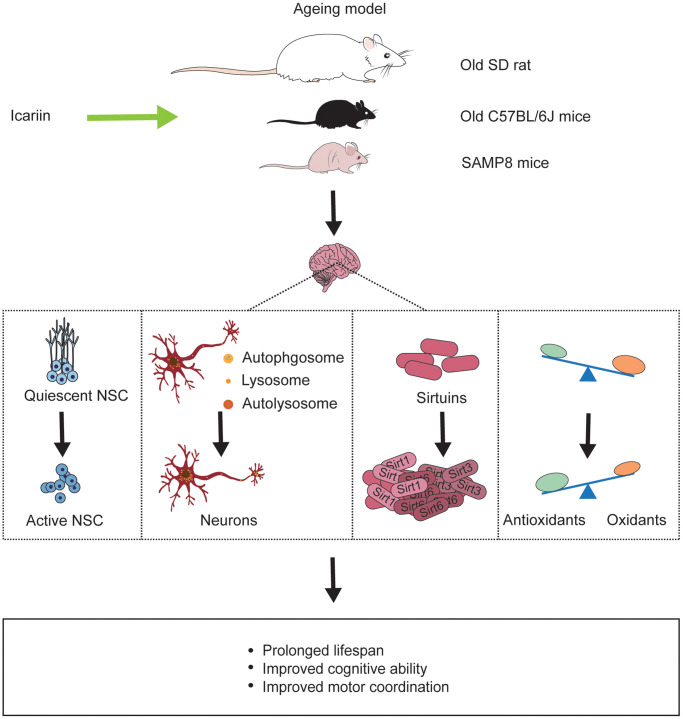
**Icariin reverses ageing-induced deficits in the brain of ageing models.** Icariin not only prolongs life span of ageing animals but also improves cognitive function and motor coordination by promoting neurogenesis, enhancing neuronal autophagy, increasing sirtuins protein like Sirt1, Sirt3, Sirt6, and exerting antioxidant effect in the brain.

Ageing, a common physical progress with decreased regeneration, body resistance, and increased disease susceptibility [102], causes physical changes like skin pigmentation, body contraction, organ failure. Ageing has become an issue of growing concern globally, and it is crucial and necessary to find new therapeutic guidelines to fight ageing. In addition, the new approaches should not only aim to prolong the life expectancy but also keep a fit body [103]. In ancient China, many emperors sought immortality by taking elixir, but they all failed. Today, increasing evidences indicate that some herbs can delay ageing [104]. Icariin produces significant beneficial effects in both *in vitro* and *in vivo* models of ageing. It may reduce cell senescence and improve the spatial memory in SAMP8 model [105] (Figure 3).

Ageing is often accompanied by neurodegeneration, and there are some similar mechanisms between ageing and neurodegeneration, such as oxidative stress, neurogenesis, and neuroinflammation. Therefore, it is reasonable to assume that preventing and delaying ageing may also slow down the occurrence and development of neurodegeneration. The brain may be the most vulnerable organ in the whole ageing process of the human body due to its high oxygen consumption and low antioxidant capacity [106]. Brain ageing decreases memory ability and cognitive function and increases senescent cells and neuronal loss [107]. Icariin reduces the impairment of the spatial memory in the SAMP8 mouse model. Senescence-associated β-galactosidase (SA-β-gal) staining demonstrated that icariin could delay the ageing progress of brain cells [105]. The hippocampus acts an important role in cognitive function [108]. The rate of neurogenesis in the hippocampus declines with age, and this decline has been closely associated with cognitive decline in mammals and humans [109, 110]. Icariin improves cognitive function in natural ageing rats via a potential mechanism associated with activating quiescent neural stem cells [111].

SIRTs are nicotinamide adenine nucleotide (NAD+)-dependent deacetylases, which participate in the cell cycle, gene repair, metabolism, and oxidative stress [112, 113]. In yeast, *Drosophila, Caenorhabditis elegans (C. elegans)*, mice, and human beings, Sirts are highly expressed and related to lifespan extension [114–116]. In old (24-month-age) male C57BL/6J mice, icariin increased the decline of Sirt 1, 3, and 6 proteins induced by ageing in the brain [117].

In addition, several studies have proved that ageing is accompanied by dysfunctional autophagy [118]. Icariin reduces neuronal dysfunction in ageing rats by promoting neuronal autophagy through the AMPK/mTOR/ULK1 pathway [119]. Icariin could ameliorate the motor and learning disabilities of male C57BL/6J ageing mice by activating Akt/Nrf2/NF-ĸB signaling pathway [117].

## Pharmacodynamics of icariin after radiation exposure

Human beings are exposed to natural radiation, i.e., terrestrial radioactivity and indoor radon, at an annual average individual effective dose of about 2.4 mSv and artificial radiation. The latter includes X-rays from X-ray machines, computed tomography (CT examination), and positron emission tomography (PET scan) (PET/CT scan) for medical diagnosis and radiotherapy [120, 121]. Exposure to high dose/dose radiation rates may cause different cancer and non-cancerous diseases such as cataract, atherosclerotic, cardiovascular, cerebrovascular, and neurodegenerative diseases [122]. Low dose/dose rate radiation may also cause damage to the human body, inducing cancer [121, 123], cardiovascular disease [124], cataract [125], neuropsychiatric disorders [126]. Nowadays, radiotherapy is a universal treatment for the brain and head-and-neck cancers [127] but with side effects such as impairments of cognition, language acquisition, and visual spatial ability [128, 129], in particular, in young patients as the developing brain is more radiosensitive [128–130].

Amifostine/ethyol, 2-(3-aminopropyl) aminoethylphosphorothioate is the only radioprotective drug approved by the US Food and Drug Administration (FDA). It is used as a reliever for xerostomia after radiotherapy for head and neck tumors [131]. However, due to its side effects such as vomiting, drowsiness, hypotension, and limited administration mode (currently only approved for intravenous administration), the clinical use of amifostine is very limited [132, 133]. Moreover, amifostine is inactive in the brain [134]. Therefore, it is imperative to find a novel radio-neuro-protective drug to prevent irradiation-induced brain injury.

Icariin protects radiosensitive organs such as reproductive and digestive organs from radiation damage and has radiosensitization effect on some cancer cells. In a ^60^Co-γ-ray-induced mouse model of spermatogenic disturbance, icariin combined with lycium barbarum polysaccharide and resveratrol promoted spermatogenesis and sperm motility [135]. *In vitro* study indicated that icariin treatment improved the radiosensitivity of colorectal cancer cells by inhibiting NF-ĸB signaling [136]. Similarly, icaritin, a hydrolysate of icariin, also synergistically enhanced the radiosensitivity of 4T1 breast cancer cells [137]. Icariin and icaritin have also been reported to inhibit UVB-induced photoaging by activating Nrf2/ARE signaling, suppressing JNK’s and ERK’s phosphorylation as well as NF-ĸB’s expression [138]. In a recent study, icariin was found to significantly increase bone marrow mesenchymal stem cells (MSCs) regeneration and repair pancreatic injury in L-arginine/γ-ray-caused rat model of chronic pancreatitis [139].

In the BALB/C mouse model, epimedium extract treatment reduced acute radiation-induced impairment of neurogenesis in the subgranular zone (SGZ) of the dentate gyrus (DG) and improved animal learning and memory ability. Moreover, epimedium treatment prevents radiation-induced weight loss, depression, and spatial memory impairment [140]. Icariin has been found to ameliorate radiation-induced toxicity in the intestine and testis of male C57BL/6 after whole body radiation by X-ray [141]. Therefore, it appears that icariin may be a candidate drug to protect against radiation-induced injury (Table 1). Given the limited research data, future study on radio-neuro-protective effect of icariin is still needed.

**Table 1:** Radioprotective effect of icariin.

**No.**	**Model**	**Radiation type and dosage**	**Drug and dosage**	**Dosing period**	**Effect**	**Mechanism**	**References**
1	Mouse model of spermatogenic disturbance induce by ^60^Coγ-ray	γ-ray; 6 Gy	Icariin, Lycium barbarum polysaccharide and resveratrol; 80 mg/kg respectively	60 days	Promoted spermatogenesis and sperm motility	Did not mention.	[126]
2	Colorectal cancer cell lines: HCT116 and HT29; Xenograft Mouse Model	X-ray; 0, 2, 4, 6 Gy *in vitro* and 4 Gy *in vivo*	Icariin; 25 uM *in vitro* and 40 mg/kg *in vivo*	4 h *in vitro* and 3 weeks *in vivo*	Enhanced the radiation-mediated anti-proliferative effect; exerted the anti-proliferative and/or pro-apoptotic effect possibly	Inhibited the activation of NF-ĸB signaling pathway	[127]
3	Murine 4T1 breast cancer cells	IR; 0, 1, 4, 6, 8Gy	Icaritin; 0, 1.5, 3, 6, 12.5, 13, 25 uM	4, 24, 48, 72 h	Exert an anti-proliferative effect; induce the G2/M blockage of 4T1 cells; Synergize with IR to enhance 4T1 cell apoptosis	Suppressed the activation of ERK1/2 and AKT signaling pathways	[128]
4	UVB-irradiated human keratinocytes (HaCaTs)	UVB; 125 mJ/cm²	Icariin and icaritin;1, 10, 100 nM respectively	24 h	Produced anti-oxidative stress, anti-inflammation and anti-photoageing effects	Promoted Nrf2/ARE signaling; inhibited JNK and ERK phosophorylatio; suppressed NF-ĸB expression	[129]
5	Chronic pancreatitis rat model induced by L-arginine/radiation	γ-ray; 6 Gy	Icariin; 100 mg/kg	8 weeks	Promoted MSCs proliferation and differentiation; Synergize with MSCs to improve the function of pancreatic stellate cells	Did not mention	[130]
6	X-ray-irradiated BALB//C mouse model	X-ray; 5.5 Gy	Epimedium extract; 5 g/kg	4 weeks	Improved animal weight loss, locomotor activity and spatial learning and memory		[131]
7	X-ray-irradiated C57BL/6 mice	X-ray; 4 and 7 Gy	Icariin; 10, 20, 40 mg/kg	24 h and 30 min before and 24 h after irradiation	Protected the radiosensitive organs such as intestine, testis and hemopoietic system	Produced effect partly through its anti-oxidative and anti-apoptotic properties	[132]

## Molecular mechanisms of the effect of icariin in brain

### Icariin-mediated autophagy-related effects

Some environmental toxicants may induce PD, especially pesticides. Rotenone (ROT) is one of the pesticides with neurotoxicity that affects the normal course of autophagy [142, 143] and has been considered to be related to the genesis or development of PD and reduced DA neurons in PD [144]. In a rat PD model induced by ROT, icariin significantly reduced neurotoxicity by activating autophagy in the brain [144]. Intra-cephalic Aβ accumulation has been related to AD development [21, 145]. Aβ produced in endonuclear and autophagic vacuoles may be effectively transmitted to other organelles by lysosomal proteolysis. In AD, the dysfunction of autophagic vacuoles or lysosomal proteolysis induces Aβ deposition in the brain [146]. In addition, the formation of elevated Aβ deposition is caused by inhibition of basal autophagy, resulting in neurodegeneration [142]. Hence, the regulation of autophagy dysfunction may be a good strategy for AD patients. Icariin reduces the cognitive impairments and autophagic dysfunction in the rat AD model induced by intracerebroventricular (icv) injection of Aβ1-42 [143]. Mechanistic studies have shown that icariin reduce oxygen-glucose deprivation and reperfusion (OGD/R)-induced high level of autophagy by increasing the level of Bcl-2 and decreasing the Beclin-1 and LC3-II levels in OGD/R-treated PC12 cells [147]. In a rat model of natural ageing, icariin improves autophagy in the brain by regulating AMPK/mTOR/ULK1 signaling pathway.

### Anti-apoptotic effect of icariin

Excitotoxicity causes neuronal death and is involved in the development of AD [24]. Neurodegeneration is an important pathological characteristic of AD [148]. Intracerebroventricular injection of excitatory neurotoxin ibotenic acid triggers caspase family and causes apoptosis leading to irreversible damage in the hippocampus [149]. Icariin significantly increases pro-caspase-3 expression and decreases active-caspase-3 in the rat ibotenic acid-induced excitotoxicity model, suggesting its neuroprotective effect by reducing neuronal apoptosis [24]. Moreover, in the APP/PS1 transgenic mice model, icariin reduces apoptotic cells induced by endoplasmic reticulum (ER) stress through suppressing the protein kinase RNA-like ER kinase/eukaryotic initiation factor 2α (PERK/Eif2α pathway) in the hippocampus [150]. *In vitro* study indicates that icariin protects hypothalamic neurons from apoptosis by the activation of PI3K/Akt signaling pathway [79]. These results are consistent with the previous reports that icariin inhibits CORT-caused rat hippocampal neuronal apoptosis by inhibiting the p38 MAPK signaling pathway [78], and suppresses ER stress-induced neuronal apoptosis by activating the PI3K/Akt signaling pathway [151]. In PC12 cells model of ER stress, anti-apoptotic effects of icariin may partially be associated with the up-regulation of synoviolin, an essential anti-apoptotic factor that inhibits cell death caused by ER stress [152].

Hydrogen peroxide (H_2_O_2_)-induced oxidative stress may cause apoptosis by activating JNK/p38 MAPK pathways [153]. p53, a transcription factor, may be associated with neuronal apoptosis regulation [154]. The neuronal apoptosis will start when p53 is activated, and p53 activation is closely related to the JNK/P38 MAPK pathway. In addition, JNK/p38 MAPK from MAPKs family is related to the anti-oxidation process. It may serve as an important target for treating oxidative stress-induced neurodegenerative diseases [155]. Activation of JNK/p38 MAPK induces cellular apoptosis [156]. Under H_2_O_2_ exposure, phosphorylation of JNK/p38 MAPK occurs in PC12 cells. Pretreatment of icariin reduces the number of neuronal apoptosis by inhibiting the phosphorylation of JNK/p38 MAPK pathway, suggesting that neuroprotective effect of icariin may be mediated through inhibition of JNK/p38 MAPK signaling pathway [157].

Akt, also known as protein kinase B, is the crucial downstream factor of PI3K. The phosphorylation of Akt plays a vital role in the PI3K/Akt signaling pathway [158, 159], it regulates various substrates, such as GSK-3β, Bcl-2 [160] and is involved in the pathogenesis of different brain diseases, including AD [161], epilepsy [162], and cerebral ischemia [163]. Activation of PI3K signaling prevents many apoptosis stimuli [164]. In PD animal model induced by MPTP, icariin effectively protects mouse from neurotoxicity and reduce the occurrence of neuronal apoptosis in the substantia nigra accompanied by decreased Bcl-2, increased Bax, and caspase 3 protein expressions, as well as the increased phosphorylation levels of Akt. In addition, these neuroprotective effects may be blocked by LY294002 (a PI3K inhibitor) [50], which indicates that the PI3K/Akt signaling pathway may contribute to icariin’s anti-apoptotic effect. The anti-apoptotic effect of icariin may also be regulated by the inositol-requiring enzyme-1/X-box-binding protein-1 (IRE1/XBP1) pathway in the OGD/R cell model [165].

### Anti-oxidant effects of icariin

Oxidative stress is an imbalance in which the production of reactive oxygen species in the body far exceeds the body’s anti-oxidant capacity [106]. When oxidative stress occurs, lipid peroxidation damages the cell membrane, induces irreversible changes in the structure and function of some proteins (e.g., structural proteins and enzyme proteins), and DNA damage [106]. The brain is an organ that needs high energy and high oxygen due to its abundant peroxidable polyunsaturated fatty acids, high concentration of reactive oxygen catalyst iron, relative lack of anti-oxidant enzymes, and susceptible to oxidative stress [166].

Oxidative stress induced by H_2_O_2_ is an important contributor to the pathogenesis of AD [157]. Hydrogen peroxide belongs to the high reactive oxygen species (ROS). It causes neuronal apoptosis by activating relevant molecular pathways when oxidative stress is induced [153, 167]. Icariin prevents hydrogen peroxide-induced PC12 cells death, suggesting that it has an anti-oxidant effect [157]. The anti-oxidant effect of icariin to H_2_O_2_-induced cytotoxicity may be related to the up-regulation of SIRT1 [168]. Moreover, icariin may reverse the upregulation of MDA and downregulation of SOD activity in 2-VO rats, leading to neuroprotection [61]. In an unpredictable CMS rat model of depression, icariin produces antidepressant-like effect partially by mediating the anti-oxidation process [61]. In AD mouse model, icariin attenuates excess iron-induced brain oxidative stress [61]. In addition, icariin partially reverses learning and memory impairments induced by aluminum, which may also be associated with its anti-oxidant effect [41]. *In vitro* study indicates that icariin may decrease ROS production in LPS-treated microglia [85].

Nrf2 regulates important genes, such as promoter regulatory regions, enabling cells to maintain a stable situation from oxidative stress, inflammation, and biotransformation [169]. Nrf2 is considered a crucial transcription factor that modulates cellular redox state in the case of oxidative stress [170]. Moreover, substantial evidence indicates that loss of Nrf2 may be involved in the development of neurodegenerative diseases. In addition, Nrf2 inactivation has been associated with ageing and PD [171]. In the 6-OHDA-induced neurotoxicity of PC12 cell model, icariin attenuates the accumulation of reactive oxygen species and improve the survival rate by introducing more active Nrf2. These results suggest that icariin may serve as an anti-oxidant by the activation of Nrf2 pathway [19].

### Anti-neuroinflammatory effects of icariin

Microglia-mediated neuroinflammation plays a key part in the pathogenesis of neurodegenerative diseases. Accumulating evidences show that inhibition of neuroinflammation may effectively mitigate neurodegenerative diseases [172]. Neuroinflammation is characterized by the activation of glial cells, especially microglial activation leading to a significant increase of inflammatory cytokines and chemokines [173]. Icariin inhibits glia-mediated neuroinflammation by activating Nrf2 signaling in both *in vitro* and *in vivo* models [85, 172]. In the mouse models of LPS-induced hippocampal neuroinflammation and social defeat, icariin exerts an anti-neuroinflammatory effect by inhibiting HMGB1-receptor for advanced glycation end products (RAGE) signaling [65, 174]. In CUMS-induced rat model of depression, icariin produces anti-neuroinflammatory and anti-depression effect partially by the inhibition of NF-ĸB pathway and the NLRP3-inflammasome/caspase-1/IL-1β axis [28]. In APP/PS1 transgenic mice, icariin also reduces neuroinflammation induced by microglial activation in the cortex [175]. Icariin may also reduce neuroinflammatory response in LPS-stimulated microglia by suppressing the TAK1/IKK/NF-κB and JNK/p38 MAPK signaling [85]. In addition, icariin protects DA neuronal damage induced by LPS and 6-OHDA, which is partially regulated by its suppression of microglia-mediated neuroinflammation [52].

### Icariin promotes neurogenesis

In the mammalian brain, the process by which neural stem cells (NSCs) proliferate and differentiate into new cells such as astrocytes, neurons, and oligodendrocytes is called neurogenesis [176–178]. Adult neurogenesis mainly occurs in discrete brain areas such as the SGZ of the DG and the subventricular zone (SVZ) adjacent to the lateral ventricles [179, 180]. Decreased neurogenesis in the hippocampal DG region of AD animal models has been well documented [181–184]. Promoting neurogenesis may significantly improve hippocampal-dependent cognitive function [185–188]. In Tg2576 mice, Icariin promotes hippocampal neurogenesis and improves memory ability [26]. In SD rat model of cerebral ischemia, icariin and mesenchymal stem cells (MSCs) synergistically improve neurogenesis [189]. *In vitro* studies show that icariin promotes human NSC proliferation by regulating the various related genes [190] and the growth and proliferation of NSCs in cultured rat hippocampus by regulating the levels of cyclin D1 and p21, which exert an important effect in modulating cell cycle [191]. NSCs are divided into quiescent NSCs and active NSCs [192], and the activation of quiescent NSCs increases neurogenesis. In the rat ageing model, icariin induces the transformation of quiescent NSCs into active NSCs [111]. It enhances the proliferation, viability, and migratory ability of NCSs *in vitro*, promotes their differentiation into neurons *in vivo* [193] and facilitates the self-renewal of NSCs by regulating ERK/MAPK signaling pathway [152].

### Icariin produces neuroprotective effects by modulating various other signal transduction pathways

SIRTs are a small family of proteins, comprising of SIRT1-SIRT7 [30]. In ageing and other pathological processes, SIRTs are involved in regulating transcription, cell cycle, cell differentiation, apoptosis, stress, metabolism, and genome stability [194]. Among family members, SIRT1 is an important molecule that has been associated with neuronal stress response [63]. Under ischemia/reperfusion circumstances, SIRT1 may protect cortical neuronal damage by inhibiting p53 [195]. Moreover, SIRT1 extends lifespan through increasing gene stability in lower organisms such as yeast and mammals [196]. In addition, SIRT3, another important member of SIRTs, which exists in mitochondria, regulates mitochondrial function by modifying protein in many tissues including the brain [197]. SIRT3 protects neurons from an oxidative stress injury in mitochondria and prolongs the lifespan of the neurons [198]. SIRT3 is related to neurodegenerative diseases such as AD, PD, HD [199]. Moreover, in neurons, PGC-1 family is a powerful stimulator of mitochondrial respiration and gene transcription [200]. It has been reported that PGC-1α, a member of PGC-1 family, regulates mitochondrial function [201] by inducing mitochondrial gene expression and mediating energy metabolism [202]. Additionally, elevated PGC-1α level reduced neuronal death caused by oxidative stress [203]. In rats and PC12 cells, the expression of PGC-1α reduces with the decrease of SIRT3 after ROT treatment [199]. In addition, ICA alleviates ROT-induced oxidative stress toxicity by modulating mitochondrial antioxidant SOD2 through increasing the levels of SIRT3 and PGC-1α [199]. Overexpression of SIRT1 activates PGC-1α to regulate neuronal metabolism and mitochondrial function [200, 204, 205]. Icariin produces neuroprotective effects in ischemic brain injury [63]. In stroke model, PGC-1α increases parallel to the SIRT1 after icariin treatment. The neuroprotective effect of icariin could be reversed by a SIRT1 inhibitor [63]. Therefore, the activation of SIRTs-PGC-1α signaling pathway may contribute to the neuroprotection of icariin.

ERK is actively involved in the Ras-Raf-ERK pathway and related to cell development and proliferation [206]. In the mammalian brain, ERK may be involved in the process of dendritic protein synthesis, which plays a vital role in memory and long-term potentiation (LTP) formation [207]. Ca^2+^/calmodulin-dependent protein kinase II alpha (CaMKIIα), a protein kinase with multiple functions, is highly expressed in the hippocampus. In terms of learning and memory and LTP in particular, CaMKIIα plays a significant role [208]. CREB is a downstream protein of CaMKII, the transcription of CREB and CaMKII is conducive to neuronal plasticity and development. The impaired learning and memory function induced by chronic multiple-stress is improved through the elevation of CREB and CaMKII mRNA expression in the brain [208]. In prenatally stressed female offspring, reduced spatial learning and memory and working memory ability are demonstrated by MWM test and 8-Arm Maze. These animals also show downregulated expression of CaMKIIα and CREB in the hippocampus [209]. Treatment with icariin improves animal learning and memory and upregulates hippocampal CaMKIIα, CREB, and ERK [209]. These results strongly suggest that icariin can effectively alleviate the influence of prenatal stress on rat offspring by the activation of ERK/CaMKIIα/CREB pathway.

**Figure 4:**
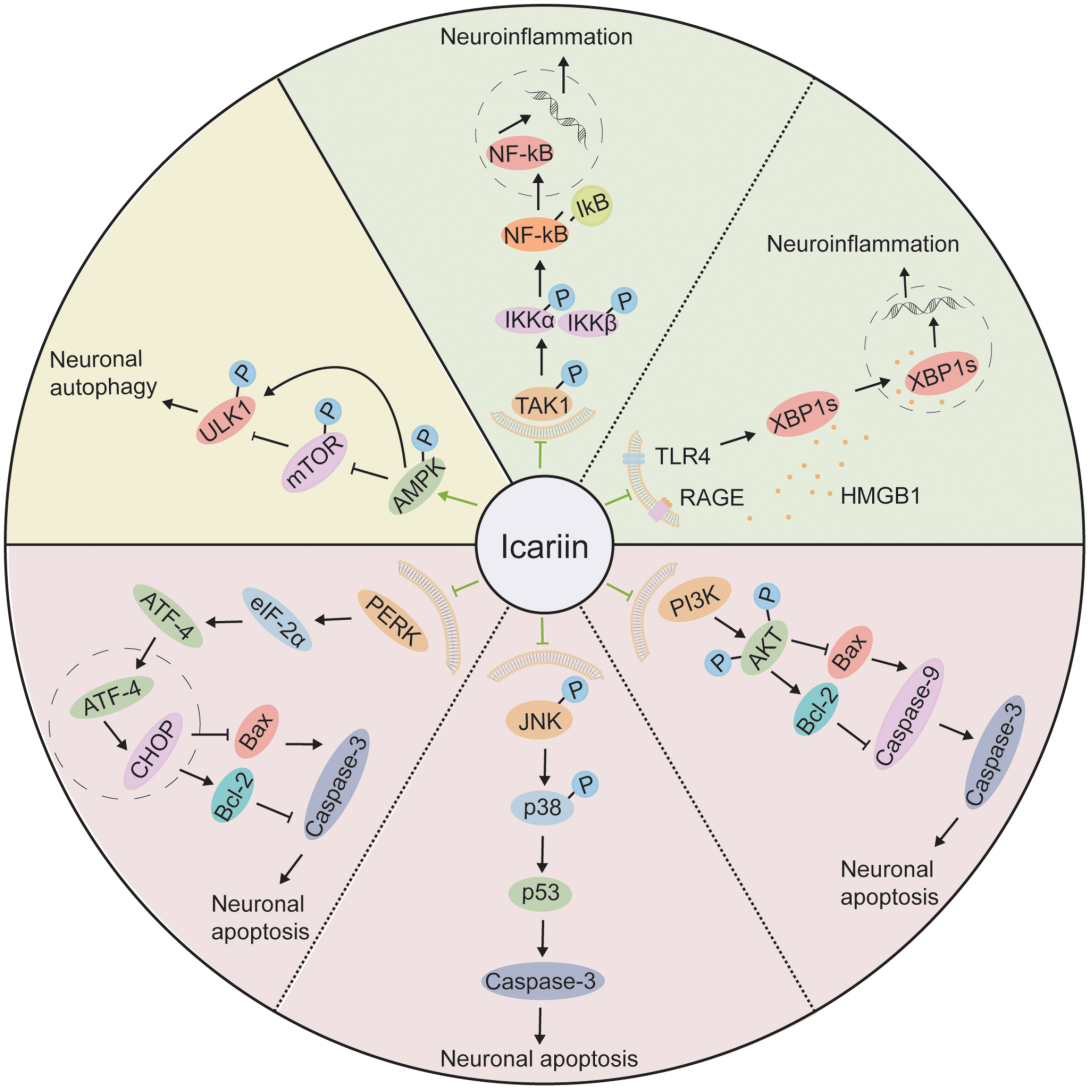
**The molecular mechanisms of the neuroprotective effect of icariin.** Icariin improves neuronal autophagy through the AMPK/mTOR/ULK1 pathway; It attenuates neuroinflammation by inhibiting TAK1/IKK/NF-κB and HMGB1/RAGE pathways; It reduces neuronal apoptosis by suppression of PERK/eIF2α, JNK/p38 MAPK, and PI3K/AKT pathways.

The detailed molecular mechanisms underlying icariin’s neuroprotective effects have been indicated in Figure 4.

## CONCLUSIONS AND FUTURE STUDIES

Animal experimental studies have strongly suggested the neuroprotective effects of icariin on neurological and neuropsychiatric diseases, brain ageing, and injury induced by radiation exposure. These neuroprotective effects are mainly mediated through regulation of neuroinflammation, neuronal apoptosis, and autophagy. Recent studies have indicated that icariin may also be used to treat brain diseases such as prenatal stress-induced offspring cognitive impairment [209], demyelinating disease [210], and MS [99]. An icariin-NGSTH (nanogel loaded self-assembled thermosensitive hydrogel) has been developed to effectively control the depression by continuous release of icariin through adhesion of nasal mucosa *in vivo* [71]. The ethosomal gel of icariin combined with hydroxysafflor yellow A (HSYA), Epimedin B, and 3, 4-dihydroxybenzoic acid could be used to treat peripheral neuropathy caused by oxaliplatin [211]. Icariin has also been found to exert an anti-atherosclerosis effect by regulating some pathways involving lncRNA (Lon non-coding RNA) and mRNA [212], suggesting its neuroprotective roles may also be regulated by lncRNA and mRNA.

Further studies in the following areas may still be needed: 1) development of a novel delivery system in order to increase the efficiency of icariin to enter the CNS and effectively treat brain diseases; 2) understanding the roles of icariin on non-coding RNAs such as microRNAs, lncRNAs, in order to target them to effectively prevent the genesis of different brain diseases; and 3) investigation of the effect of icariin on gut microbiota so as to reveal novel mechanisms of icariin on gut-brain-axis, and its therapeutic effects on the different neurological, neuropsychiatric disorders and brain radiation exposure.
